# The Natural *Antherea pernyi* Sericin Protein Suppresses Gastric Cancer Formation by Inhibiting Cell Proliferation and Inducing Cell Apoptosis

**DOI:** 10.3390/ijms26051890

**Published:** 2025-02-22

**Authors:** Qiong-Qiong Wei, Yu Xiao, Qiao Wu, Cai Jing, Zhan-Qi Dong, Peng Chen, Min-Hui Pan

**Affiliations:** State Key Laboratory of Resource Insects, Key Laboratory of Sericultural Biology and Genetic Breeding, Ministry of Agriculture and Rural Affairs, Southwest University, Beibei District, Chongqing 400715, China; qiongqiongwei@126.com (Q.-Q.W.); xiaoyu8230@126.com (Y.X.); wuqiao575757@163.com (Q.W.); jingcai0218@163.com (C.J.); zqdong@swu.edu.cn (Z.-Q.D.)

**Keywords:** gastric cancer, sericin, silkworm, apoptosis, cisplatin

## Abstract

Sericin, a natural macromolecular protein and the main component of silkworm cocoons, exhibits biocompatibility, excellent mechanical properties, and biodegradability. Previous research has confirmed that the sericin protein possesses anticancer properties. Gastric cancer (GC) poses a serious hazard to human health, with a low rate of early diagnosis and a poor prognosis. Investigating the safety and effectiveness of drugs for their used in treatment is imperative. In this study, we confirmed that *Antherea pernyi* sericin (APS) inhibited the proliferation, migration, and clonal formation of GC cells and caused apoptosis in the cells by regulating the expression of Bcl2 and Bax. Moreover, our data show that APS did not exhibit significant toxicity in normal gastric mucosal cells and mice. Furthermore, the results show that APS suppressed the proliferation of cisplatin-resistant GC cells and promoted cellular apoptosis; however, it had no synergistic effects with cisplatin. All the results indicated that APS exhibits antitumor activity against GC and is a prospective medicinal agent for the clinical treatment of GC, with minimal toxicity and adverse side effects. This research can provide a theoretical basis for sericin in the field of tumor treatment, especially for the application of natural macromolecular polypeptide drugs.

## 1. Introduction

Gastric cancer (GC) is a malignant and lethal tumor, the incidence and mortality of which rank fifth in the world [1]. Although the worldwide incidence rate of GC has de-creased over time [2,3], it still has the highest mortality rate and poses a serious threat to human health. In recent years, there have been some achievements in the comprehensive treatment of GC. Surgery combined with neoadjuvant chemotherapy can effectively prolong the survival time of patients [4]. Targeted therapy and immunotherapy are currently the main treatment methods for GC, such as HER2-targeted drugs, VEGF inhibitors, and PD-1/PD-L1 inhibitors, but drug resistance and side effects are still difficult problems in the clinical treatment of GC. Therefore, screening drugs with diversified treatment such as anti-tumor activity and the ability to reverse tumor resistance is a current priority to provide patients with a more effective, low-toxicity, comfortable, and economical comprehensive treatment.

Peptides are compounds formed by the dehydration of three or more amino acids. They are derived from widespread sources, including animals, plants, bacteria, and fungi. Peptides are characterized by their high activity, safety, and ease of absorption [5]. Researchers have found that they also have antitumor activity. Antitumor peptides can exert their antitumor effects by activating the body’s immune system, arresting the cell cycle, promoting cellular apoptosis, and inhibiting the formation of tumor neovascularization [6,7,8,9]. Oncolytic peptides (OPs) are peptides that selectively target cancer cells while sparing normal cells. These peptides can alter membrane dynamics and increased cellular activity, and their effects on the viability of cancer cells have been asssessed. Among them, the oncolytic peptide LTX-315 has played an important role in anti-tumor therapy [10,11]. In GC, ANXA1-derived peptides bind to EphA2 and target EphA2 for degradation, thereby inhibiting mouse gastric and colon cancer cell proliferation [12]. Apoptin-derived peptides promote GC cells’ apoptosis, necrosis, and the reversal of cisplatin resistance by inhibiting the PI3K/AKT/ARNT signaling axis [13].

Silk cocoons are mainly made up of two natural proteins, sericin and fibroin. Compared with the fibroin protein, sericin is largely ignored in the field of sericulture and is mostly discarded in waste liquids [14]. However, with the utilization of natural resources, sericin has gradually attracted the attention of researchers. Studies have shown that sericin has high potential in beauty, healthcare, and food applications, as well as functional biomaterials [15], and it has been proven that sericin has better antioxidant, anti-ultraviolet, and antibacterial activities than fibroin [16,17]. Sericin is a pure natural complex protein, constituting approximately 15–35% of the total cocoon weight; it is composed of 17–18 different amino acids that are highly soluble in water, most of which are absorbable and utilizable by the human body [18,19]. Sericin exhibits biocompatibility, excellent mechanical properties and biodegradability, making it applicable in biomedical fields, tissue engineering, and drug delivery [15]. It has been widely used as an anti-inflammatory, antioxidant, antibacterial, anticoagulant, metabolic, anti-ultraviolet, and wound-healing agent with inhibitory tyrosinase activity) and a material in the biomedical field [20,21]. A previous report showed that sericin can prevent apoptosis of hippocampal neurons in diabetic rats by triggering the Akt signaling pathway [22]. Moreover, multiple studies have suggested that sericin has antitumor effects on a variety of tumor types, such as breast, colon, and skin cancer [23,24,25,26]. Sericin caused cell cycle arrest in the G0/G1 phase by blocking the PI3K/AKT signaling axis in breast cancer cells [24]. To date, although it has been confirmed that sericin has antitumor activity, there have been few related research reports. Most studies have focused on its auxiliary antitumor effect as a biological drug delivery material. Moreover, no studies have reported whether sericin has a therapeutic effect on GC and whether it is related to chemotherapy resistance.

In the present study, we extracted sericin from *Bombyx mori* (871 strain), *Antherea pernyi* (Yuda1 strain) and *Bombyx mandarina* (AK18 strain) cocoons and used it to treat GC cells. The detection of cell proliferation activity revealed that *A. pernyi* sericin (APS) had the best effect on MKN45 cells. Therefore, APS was used as the subsequent research object. In vitro and vivo experiments confirmed that APS had low toxicity to individuals and cells. We found that APS had exhibited antitumor activity against GC in vitro, and in vivo, inhibiting the proliferation, migration, and clonal formation of GC cells and promoting cell apoptosis. Furthermore, the results suggested that APS has an effect on cisplatin-resistant MKN45/CDDP cells; however, it has no synergistic effects with cisplatin. All of these results can provide a theoretical basis for using sericin in the field of tumor treatment, especially for the application of natural polypeptide drugs.

## 2. Results

### 2.1. Extraction and Screening of Sericin

**Figure 1:**
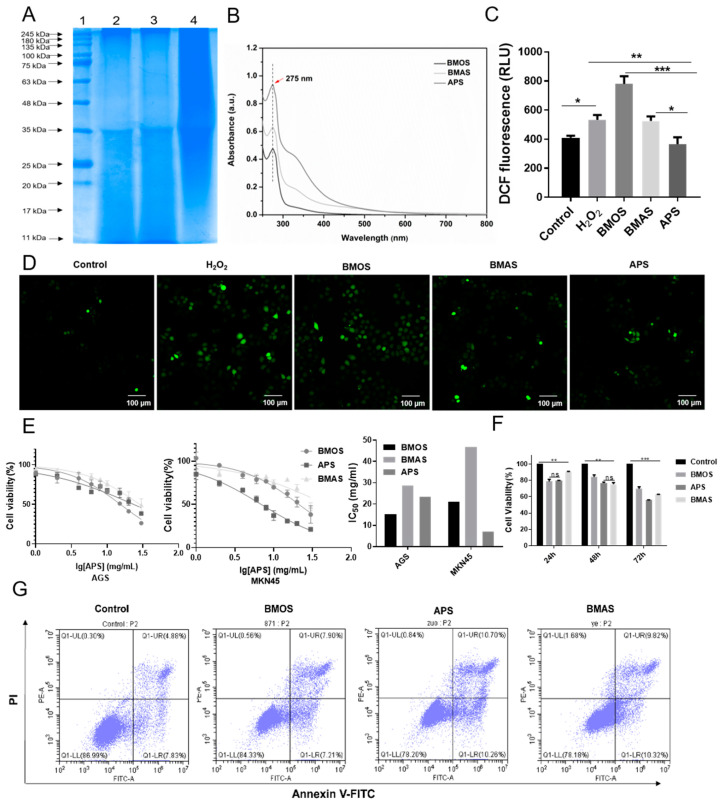
The effects of sericin from different sources on the apoptosis, proliferation, and oxidation of cells. (**A**) Coomassie bright blue staining was used to detect differences in protein size among the three kinds of sericin: 1, marker; 2, *Bombyx mori* sericin (BMOS); 3, *Bombyx mandarina* sericin (BMAS); 4, *Antherea pernyi* sericin (APS). (**B**) The ultraviolet absorption spectra of BMOS, BMAS, and APS were detected using a spectrophotometer. (**C**,**D**) The ROS levels of GES1 cells were evaluated using DCFH-DA after treating with H_2_O_2_, BMOS, BMAS, and APS for 24 h. (**E**) The IC_50_ was evaluated by performing CCK-8 tests on AGS and MKN45 cells treated with diverse concentrations of BMOS, BMAS, and APS for 24 h (1 mg/mL, 2 mg/mL, 4 mg/mL, 6 mg/mL, 8 mg/mL, 10 mg/mL, 20 mg/mL, 30 mg/mL). (**F**) The CCK-8 test was used to measure cell viability after treatment with BMOS, BMAS, and APS (4 mg/mL) or the control for 24 h, 48 h, and 72 h. (**G**) The level of apoptosis was determined using flow cytometry after AGS cells were treated with BMOS, BMAS, and APS (4 mg/mL) for 24 h. ***, *p* < 0.001; **, *p* < 0.01; and *, *p* < 0.05; and ns, *p* > 0.05.

Sericin was extracted from *B. mandarina*, *B. mori*, and *A. pernyi*, and designated as BMAS, BMOS, and APS, respectively. Coomassie Brilliant Blue staining results showed no significant differences in the protein bands of the three kinds of sericin, with clear bands at 240 kDa and 35 kDa (Figure 1A). The UV absorption spectra showed that all three types of sericin had UV absorption peaks at 275 nm, but the absorbance was different, and APS had the highest absorbance (Figure 1B), which may be attributed to differences in the amino acid composition and content of the sericin. Previous studies have confirmed that sericin has antioxidant activity [16]. Therefore, we constructed a cellular oxidation model using H_2_O_2_ (Figure S1A,B). The results of treating the cells with the three types of sericin after oxidation showed that APS reduced ROS formation (Figure 1C,D). Then, we treated GC cells with different concentrations of the sericin. CCK-8 assay results suggested that the half-maximal inhibitory concentration (IC_50_) values for BMOS, APS, and BMAS were 15.35, 23.49, and 28.58 mg/mL in AGS cells and 21.17, 6.914 and 46.7 mg/mL in MKN45 cells, respectively (Figure 1E). Further treatment of AGS cells with a sericin concentration of 4 mg/mL for 24 h, 48 h, and 72 h revealed that APS more effectively lowered cell proliferation compared to BMOS and BMAS (Figure 1F). Apoptosis assay results showed that APS significantly promoted AGS cell apoptosis (Figure 1G), and thus, we selected APS for subsequent studies.

### 2.2. Safety Evaluation of APS

**Figure 2:**
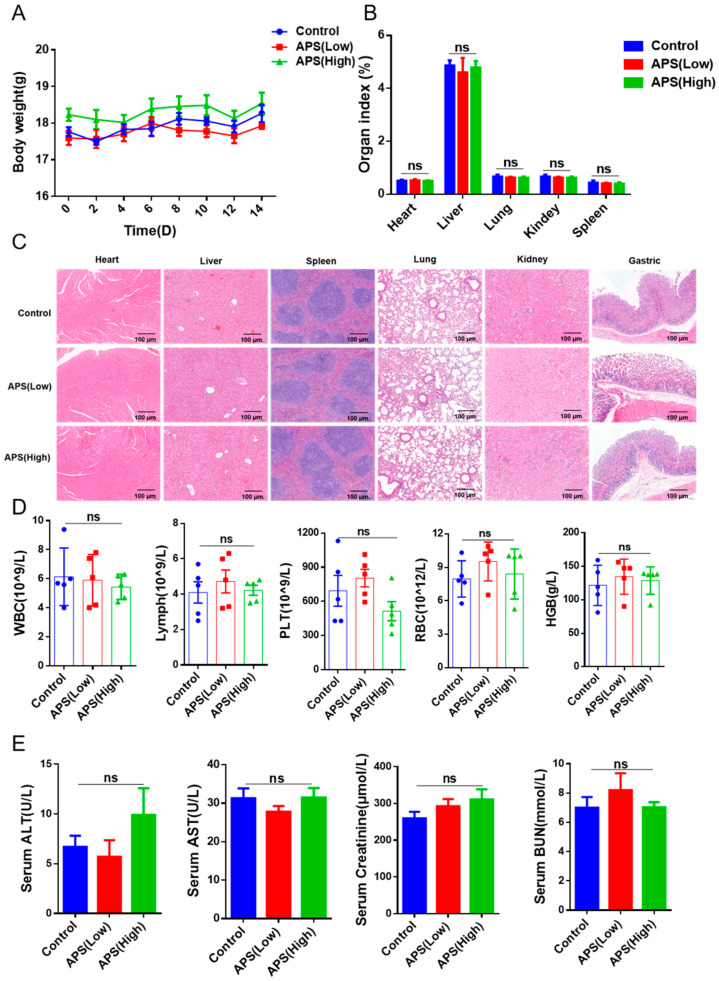
Safety evaluation of APS. (**A**) The body weights of C57BL/6 mice treated with 5000 mg/kg and 10,000 mg/kg APS via intragastric administration. (**B**) The index of vital organs, where organ index (%) = organ weight/body weight × 100%. (**C**) Representative images of hematoxylin and eosin staining of the heart, liver, spleen, lung, kidney, and stomach in the control and APS groups. Scale bar = 50 μM. (**D**) The examination of routine blood parameters, including leukocyte (WBC), lymphocyte (lymph), platelet (PLT), and erythrocyte counts (RBC) and hemoglobin (HGB), in C57BL/6 mice. (**E**) The serum alanine aminotransferase (ALT), aspartate aminotransferase (AST), creatinine, and urea nitrogen (BUN) levels of different groups. ns, *p* > 0.05.

To explore whether APS is toxic to the body, we performed a safety evaluation of APS in mice. There was no significant difference in body weight among the groups (Figure 2A). After the treatments, the heart, liver, lungs, kidneys, spleen, and stomach were collected. The organ index indicated no significant difference among the three groups (Figure 2B); differences in colon length were not significant (Figure S2A). HE staining results suggested that APS treatment had no effect on the pathological characteristics of the kidneys, liver, lungs, stomach, heart, or spleen of the mouse (Figure 2C). Blood was collected to measure routine blood parameters and biochemical indices; the data revealed no significant effects of APS treatment on routine blood indices (Figure 2D and Figure S2B), and it did not significantly affect hepatorenal function (Figure 2E). These results suggest that APS exhibits good biosafety. In vitro hemolysis experiments showed that the ddH_2_O group, the positive control, exhibited significant hemolysis, while no significant hemolysis was observed in the sericin groups at different concentrations (Figure S2C), indicating that APS has high compatibility. All of these results indicate that APS might not have any negative impacts on the mouse body and minimal toxicity.

### 2.3. APS Inhibited Proliferation, Clonogenicity, and Migration of GC Cells

**Figure 3:**
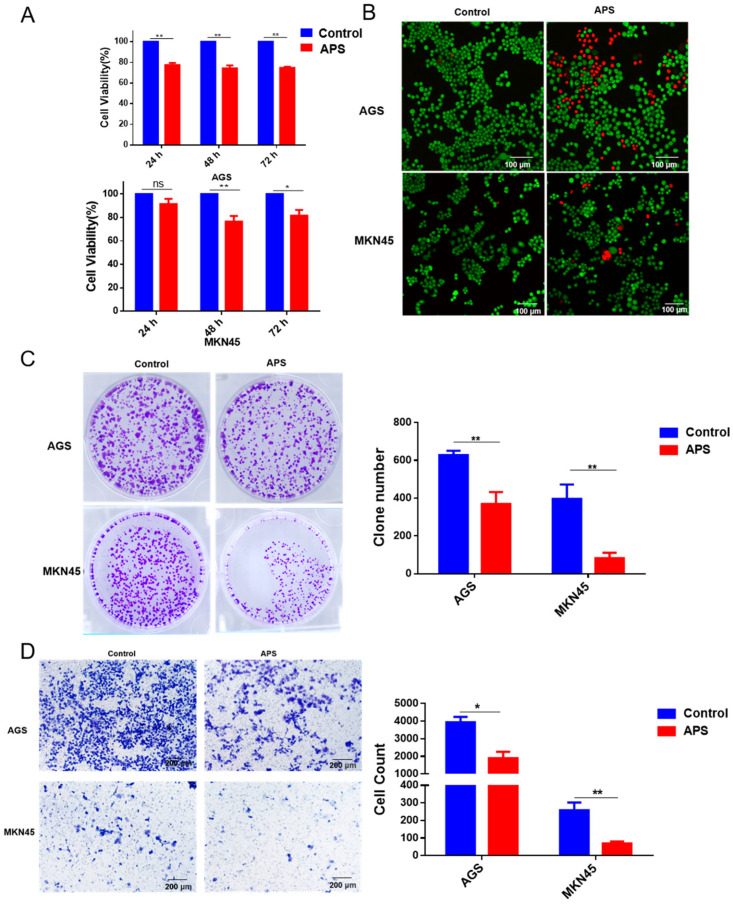
The effects of APS on GC cell proliferation, clonogenicity, and migration. (**A**) The CCK-8 test was used to measure cell viability after treatment with APS (4 mg/mL) or the control for 24 h, 48 h and 72 h. (**B**) Cells staining results of the Calcein/PI assay after treatment with APS (4 mg/mL) for 24 h. Red and green indicate dead and living cells, respectively. (**C**) The colony formation test was carried out on AGS and MKN45 cells after APS (4mg/mL) treatment. (**D**) Cell migration numbers were determined in a Transwell chamber assay after treatment for 48 h with APS (4mg/mL). **, *p* < 0.01; *, *p* < 0.05; and ns, *p* > 0.05.

To investigate the anticancer effects of APS, we treated GC cells with 4 mg/mL of APS and assessed cell viability using CCK-8 after 24 h, 48 h, and 72 h. According to the findings, APS significantly reduced the growth of GC cells compared to the medium-treated group, but there was no time-dependent trend (Figure 3A); this may be caused by the degradation of APS in the cell. In the images, where green Calcein/PI staining represents living cells and dead cells are stained red, a significant increase in dead cells after treatment with APS is observed (Figure 3B). Additionally, treatment with APS significantly reduced the clonogenicity of GC cells compared to the control (Figure 3C). It also effectively decreased the number of migrating GC cells (Figure 3D). These results demonstrate that APS can inhibit GC cell proliferation, clonogenicity, and migration.

### 2.4. APS Induces Apoptosis in GC Cells

**Figure 4:**
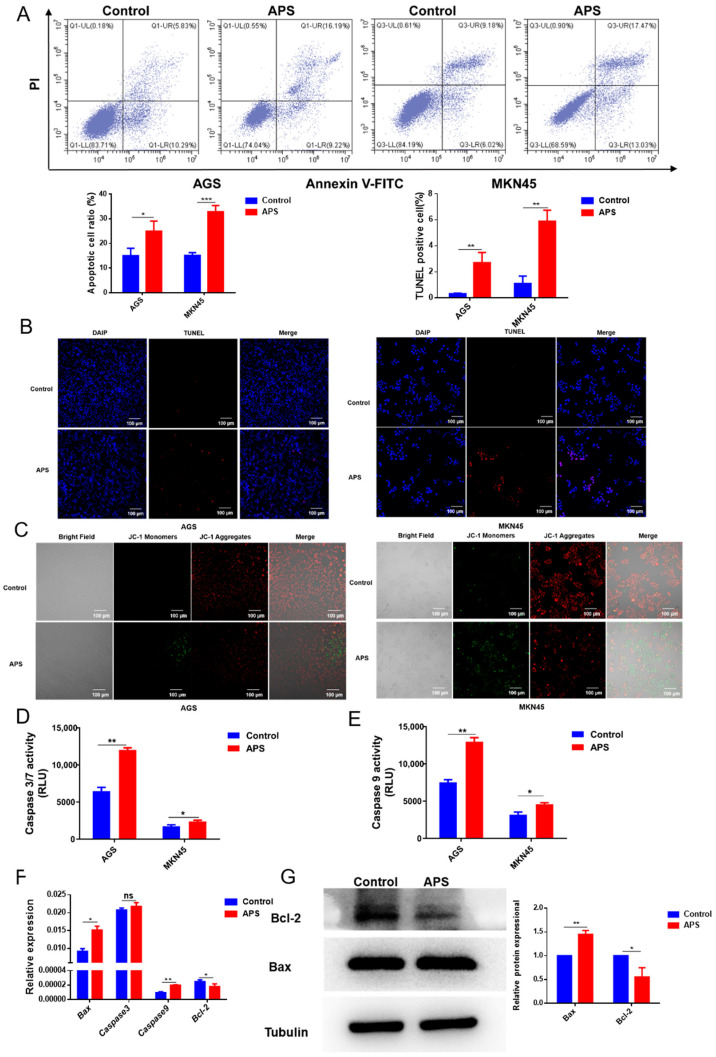
The effects of APS on GC cell apoptosis. (**A**) The level of apoptosis was determined via flow cytometry after AGS and MKN45 cells were treated for 24 h with APS (4 mg/mL). (**B**) After cell treatment with APS, the TUNEL-positive cells were identified by TUNEL staining. (**C**) The mitochondrial membrane potential was detected after AGS and MKN45 cell treatment with APS. Green fluorescence means the mitochondrial membrane potential has decreased; normally, it fluoresces red. (**D**,**E**) Caspase 3/7 and caspase 9 enzyme activities were measured to evaluate the impact of APS on apoptosis. (**F**) Quantitative real-time (qRT)-PCR analysis was used to determine the mRNA levels of *Bax*, *Caspase3*, *caspase 9*, and *Bcl2* after treatment with APS (4 mg/mL) for 24 h. (**G**) Western blotting was performed to evaluate the expression of Bcl2 and Bax after treatment with APS (4 mg/mL) for 48 h. ***, *p* < 0.001; **, *p* < 0.01; *, *p* < 0.05; and ns, *p* > 0.05.

To assess the impact of APS on apoptosis, we employed flow cytometry to determine whether treatment with APS induced apoptosis in GC cells. The results showed that the apoptotic index of GC cells following APS was substantially greater than that of the control cells (Figure 4A), although there was no discernible difference in the apoptotic cell count in normal gastric mucosal cells (GES1) after APS treatment (Figure S3A). Then, the TUNEL staining results indicated a marked increase in TUNEL-positive cells following APS treatment (Figure 4B). We assessed changes in mitochondrial membrane potential and found that it substantially decreased after treatment with APS (Figure 4C). Furthermore, caspase-3/7 and caspase-9 enzyme activities were significantly increased after treatment with APS (Figure 4D,E), whereas no changes in caspase-3/7 or caspase-9 activity were discovered in GES1 cells (Figure S3B,C). Then, we examined the expression of several crucial apoptosis-related genes using qRT-PCR, and the results showed that the expression of *Bax*, *caspase-3*, and *caspase-9* was significantly upregulated, while that of the anti-apoptotic gene *Bcl2* was downregulated after APS treatment (Figure 4F). At the protein level, the expression of the pro-apoptotic protein Bax increased, whereas that of anti-apoptotic proteins decreased (Figure 4G). All findings confirmed that APS might cause caspase-dependent apoptosis in GC cells.

### 2.5. APS Suppresses Tumorigenesis of GC

**Figure 5:**
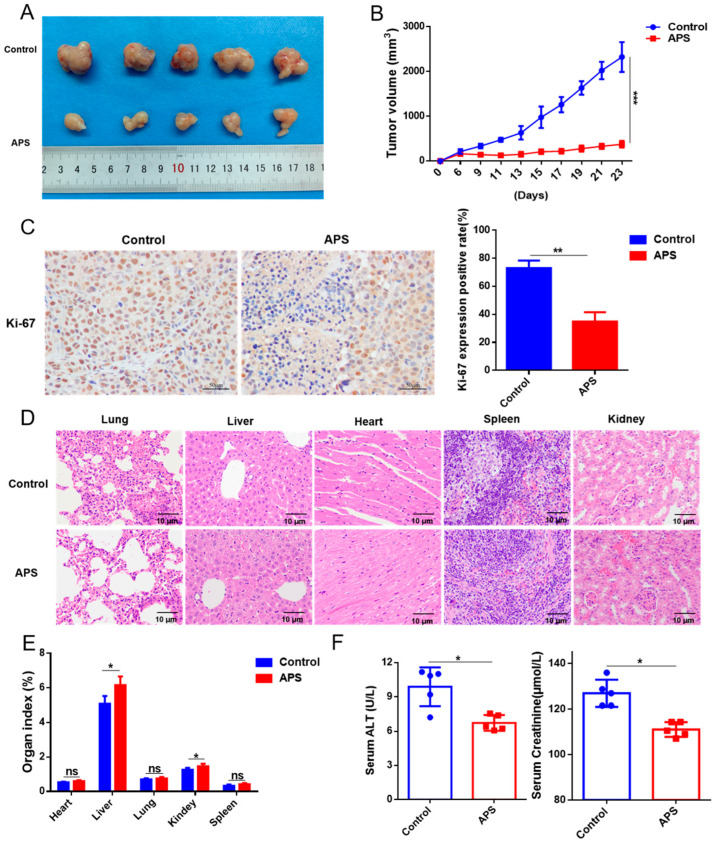
Antitumorigenic effects of APS on the GC. (**A**) Tumor xenografts were tested in MKN45 cells after treatment with APS (500 mg/kg) or ddH_2_O (control), and xenograft tumors from the indicated mice (n = 5) were imaged. (**B**) Mice in various groups had their tumor volumes measured and computed. (**C**) Relative Ki-67 intensities of the control and APS groups are presented. Scale bar = 10 μm. (**D**) H&E staining test of the heart, liver, spleen, lung, and kidney in mice in the control and APS groups. Scale bar = 10 μm. (**E**) The vital organ index. (**F**) Serum markers of liver and kidney function: ALT and creatinine. ***, *p* < 0.001; **, *p* < 0.01; and *, *p* < 0.05; and ns, *p* > 0.05.

In the tumor xenograft assay, the growth of tumors was significantly delayed with APS compared to ddH_2_O treatment (Figure 5A and Figure S4A). The tumor volume and weight of the APS group were markedly reduced (Figure 5B and Figure S4B). IHC data revealed that the expression of Ki-67 was substantially decreased in the tumor samples from the APS group (Figure 5C). We also used H&E staining to assess the effects of APS or ddH_2_O treatment on the organs of the mice. Treatment with APS appeared to alleviate the tumor’s pressure on alveolar tissue, but there were no significant pathological differences in the heart, liver, spleen, or kidneys compared to the ddH_2_O-treated group (Figure 5D). Interestingly, the organ indices indicated that the liver and kidney indices were higher in the APS-treated group than in the ddH_2_O-treated group, while there were no substantial differences in the heart, lung, and spleen indices (Figure 5E). Furthermore, we assessed liver and kidney function in the mice and found that serum AST and creatinine were substantially lower in the APS group compared to the ddH_2_O-treated group, while serum ALT and urea nitrogen showed no significant changes (Figure 5F and Figure S4C). These results suggest that APS medication may have had a protective effect on the liver and kidneys.

### 2.6. APS Inhibits Proliferation and Induces Apoptosis in Cisplatin-Resistant GC Cells

**Figure 6:**
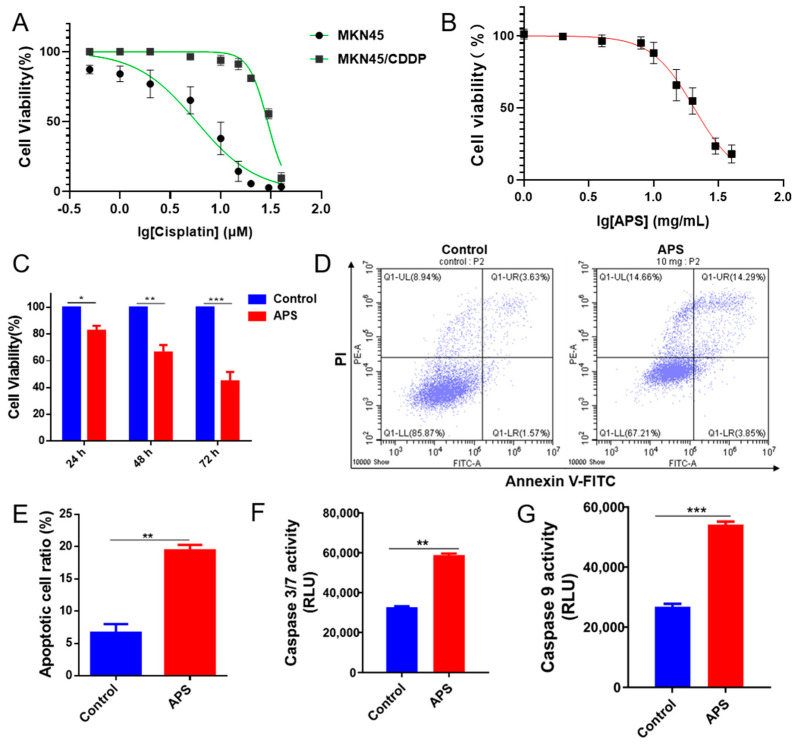
The effects of APS on apoptosis in MKN45/CDDP cells. (**A**) IC_50_ was determined by performing the CCK-8 test on MKN45 and MKN45/CDDP cells treated with different concentrations of cisplatin for 24 h (0.5 μM, 1 μM, 2μM, 5 μM, 10 μM, 15μM, 20 μM, 30 μM, and 40 μM). (**B**) IC_50_ was determined by performing the CCK-8 assay on MKN45/CDDP cells after treatment with different concentrations of APS for 24 h (1 mg/mL, 2 mg/mL, 4 mg/mL, 8 mg/mL, 10 mg/mL, 15 mg/mL, 20 mg/mL, 30 mg/mL, and 40 mg/mL). (**C**) Cell viability was tested by CCK-8 assay on MKN45/CDDP cells after APS (10 mg/mL) treated for 24 h, 48 h, and 72 h. (**D**,**E**) The apoptotic rates of MKN45/CDDP cells after treatment with APS (10 mg/mL) for 24 h, determined using flow cytometry analysis. (**F**,**G**) Caspase 3/7 and caspase 9 enzyme activities were measured to evaluate the impact of APS on apoptosis. ***, *p* < 0.001; **, *p* < 0.01; and *, *p* < 0.05.

Cisplatin, a commonly used platinum-based complex, serves as a frequently employed systemic chemotherapeutic drug for the management of GC. However, chemotherapy resistance still poses a substantial obstacle in clinical GC treatment. We hypothesized that APS could improve chemotherapeutic sensitivity. We treated MKN45 cells and cisplatin-resistant GC cells (MKN45/CDDP) with different concentrations of cisplatin, and the results showed that the IC_50_ values were 5.752 μM and 29.21 μM (Figure 6A); the IC_50_ value of MKN45/CDDP cells was significantly higher than that of MKN45 cells, indicating reduced sensitivity to cisplatin. Therefore, we treated the MKN45/CDDP cells with APS, and the results showed that the IC_50_ value was 20.51 mg/mL (Figure 6B). Treatment with 10 mg/mL APS significantly inhibited cell proliferation in MKN45/CDDP cells, showing a declining trend over time according to the CCK-8 assay (Figure 6C). The flow cytometry results indicated a significant increase in the apoptotic cell count in the APS group compared to the control group (Figure 6D,E), and caspase-3/7 and caspase-9 enzyme activities were significantly elevated (Figure 6F,G). These results demonstrated that APS can reduce the growth of MKN45/CDDP cells and accelerate cell apoptosis.

### 2.7. APS and Cisplatin Have No Synergistic Effects

**Figure 7:**
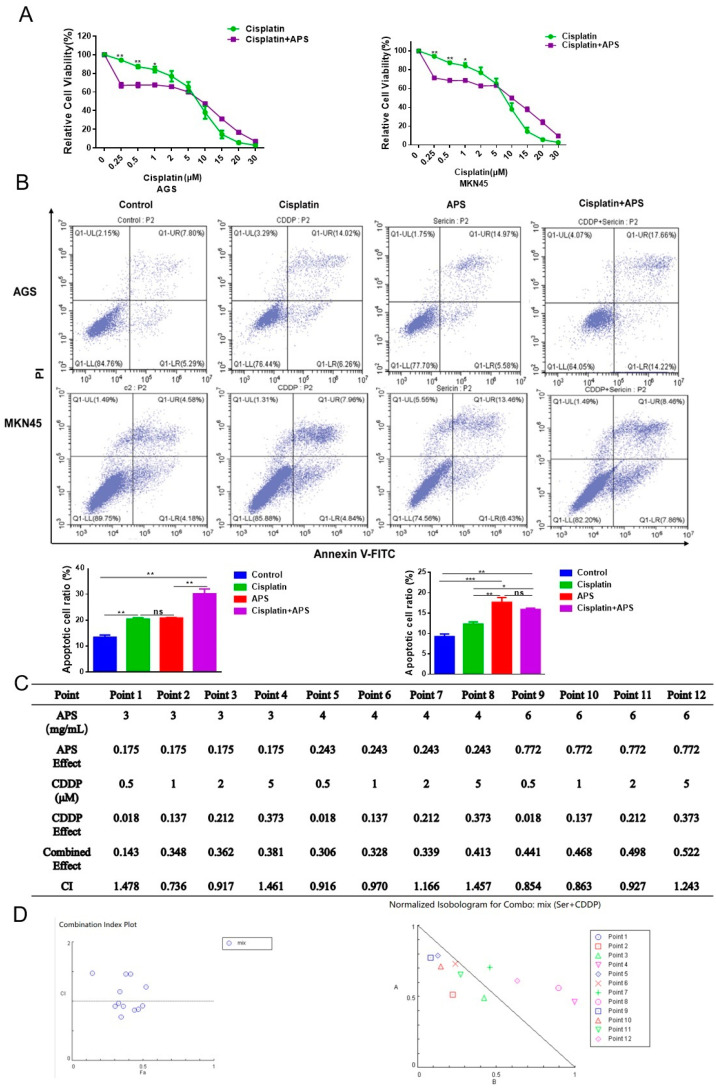
The effects of APS with cisplatin on proliferation and apoptosis in GC cells. (**A**) Cell activity was tested by CCK-8 assay after treating the cells with APS (4 mg/mL) and different concentrations of cisplatin for 24 h. (**B**) The apoptotic levels of AGS and MKN45 cells were evaluated using flow cytometry analysis after APS (4 mg/mL) or/and cisplatin (5 μM) treatment for 24 h. (**C**) APS and cisplatin concentration settings, combined effects, and CI values computed using CompuSyn software. (**D**) CI value and the combined-effect scatter plot and the dose-normalized isoeffect plots for the two drugs, with cisplatin as the ordinate and APS as the abscissa. ***, *p* < 0.001; **, *p* < 0.01; *, *p* < 0.05; and ns, *p* > 0.05.

The results above indicate that APS could prevent MKN45/CDDP cells from proliferating and cause them to undergo apoptosis. Next, we treated GC cells with varying concentrations of cisplatin and APS in order to examine whether APS could increase the sensitivity of the GC cells to cisplatin. The results showed that at low concentrations of cisplatin, the combined treatment with APS significantly reduced the growth of GC cells. Conversely, when cisplatin was combined with APS at high concentrations, cell proliferation increased (Figure 7A). Further, the flow cytometry results indicated that the rates of apoptosis were not significantly different between the synergistic treatment (5 μM of cisplatin with 4 mg/mL of APS) and cisplatin-only groups (Figure 7B). We speculated about whether APS could reduce the cytotoxicity of cisplatin in GES1 cells. However, in GES1 cells, combined treatment with APS and cisplatin produced no significant alterations in cell proliferation viability or the rates of apoptotic cells compared with the cisplatin-only group (Figure S5A,B). We investigated whether there was synergy between the two drugs by treating GC cells with different concentrations of APS and cisplatin individually or in combination. CCK-8 assays were performed to analyze cell proliferation, and the results obtained using CompuSyn3.0 software showed that the CI value in AGS cells was less than 1 when the APS concentration was 3 mg/mL with cisplatin 1 μM or 2 μM, the APS concentration was 4 mg/mL with cisplatin 0.5 μM or 1 μM, and APS concentration was 6 mg/mL with cisplatin 0.5 μM, 1 μM or 2 μM, indicating synergistic effects; however, at a cisplatin concentration of 5 μM, antagonistic effects were observed (Figure 7C,D). In MKN45 cells, the CI value was higher than 1, suggesting antagonistic action with the two agents (Figure S5C,D). These results indicate that APS has no synergistic effect with cisplatin and does not enhance the sensitivity of GC cells to cisplatin.

## 3. Discussion

GC is a multifaceted disease. Although the incidence and mortality rates of GC in China have shown a declining trend in recent years [2], less than 10% of diagnosed gastric cancer cases are diagnosed early, which is a result of factors such as the subtlety of early symptoms, health awareness, and the level of tumor screening [27]. Therefore, identifying potential anticancer materials to improve the chemotherapy sensitivity of GC cells is not only worthwhile but imperative.

Sericin, with a molecular weight of approximately 20–400 kDa, is the main component of silkworm cocoons [28]. Its variable amino acid composition and side-chain polar groups collectively endow sericin with unique properties as an antioxidant, moisturizing, healing, antibacterial, anti-UV radiation, and anti-tumor agent [21,29,30,31,32,33]. Due to the different sources of sericin, the amino acid composition and content may vary, potentially affecting its biological activity and functions [34]. Research has shown that sericin from *Philosamia ricini*, *B. mori* and *A. pernyi* can significantly downregulate TNF-α and promote the restoration of the colonic epithelial barrier following damage [35]. In this study, we treated GES1 cells and GC cells with sericin from *B. mori*, *A. pernyi*, and *B.mandarina*, and the results showed that sericin from *A. pernyi* effectively cleared ROS in GES1 cells and promoted apoptosis in GC cells. The sericin content varies significantly among *B. mori*, *A. pernyi*, and *B. mandarina*, with content at 20–30% [36], approximately 15% [37], and about 33.34%, respectively [38]. Their differing contents may lead to variations in biological functions. Meanwhile, sericin can significantly promote ROS generation in colon cancer cells, leading to cell cycle arrest in the G1 phase and apoptosis, with APS demonstrating the best effects in MCF-7 cells [23].

Previous reports have indicated that sericin can inhibit tumor occurrence in vitro [22,24,25]. According to our findings, APS successfully stopped GC cells from growing, while causing cells to undergo apoptosis. Additionally, APS showed no discernible harm to GES1 or normal animals. These results showed that APS targeted cancer cells instead of t healthy cells, which might be explained by the fact that cancer and healthy cells differ in their genomic stability [37]. One of the ways chemotherapeutic agents stop the growth of cancer cells is by apoptosis, which is a type of programmed cell death [8]. Apoptosis mainly involves two pathways: one is the intrinsic pathway related to mitochondria and several caspase-related processes, while the other is the extrinsic pathway, which involves the binding of death receptors to their ligands [39]. We found that treatment with APS promoted GC cells apoptosis, significantly increasing caspase 3/7 and caspase 9 enzyme activities, while the mitochondrial membrane potential decreased, and the expression of the anti-apoptotic protein Bcl2 was downregulated. The Bcl2 protein family primarily regulates the intrinsic pathway, which is the mitochondrial pathway [40]. These results indicate that APS leads to apoptosis in GC cells by means of the intrinsic pathway. However, this may be part of the reason why APS inhibits the proliferation of GC cells. Previous studies have reported that sericin can block the cell cycle and promote the generation of cellular ROS to inhibit the growth of tumor cells [24]; and researchers have reported that peptides can also inhibit tumor growth by regulating the body’s immune system and suppressing angiogenesis. These may also be the mechanism by which APS inhibits the proliferation of GC cells, and we can further explore this in future research.

Cisplatin is a commonly used chemotherapeutic agent for GC, and its resistance remains a major concern in clinical practice [41]. In this study, we found that APS can suppress proliferation and cause apoptosis in MKN45/CDDP cells. However, the results indicate that APS did not exhibit a synergistic effect with cisplatin. We speculated that the mechanisms of action for APS and cisplatin may differ. Cisplatin can form adducts with DNA or proteins inside the cell [42]; these DNA adducts can induce DNA damage, producing cytotoxicity that kills tumor cells [43]. On the other hand, protein adducts can induce cells to produce large amounts of ROS, which can damage the DNA double-strand backbone, further leading to DNA damage and enhancing cisplatin’s efficacy against tumor cells [44]. However, cisplatin can bind not only to tumor cell DNA but also to normal cell DNA [45], and it also has toxic side effects on the liver, kidneys, and heart [46]. Previous research on sericin mechanisms has shown that it can reduce metal ions, for example, Fe, Cu, and Zn, in normal cells, preventing metal-ion overload and clearing intracellular ROS to avoid the oxidative damage that leads to necrosis or apoptosis [15,47,48,49,50]. In tumor cells, its primary mechanism is through arresting the cell cycle and promoting oxidative stress to induce apoptosis [24]. No studies have reported that sericin can induce DNA damage in tumor cells.

**Figure 8:**
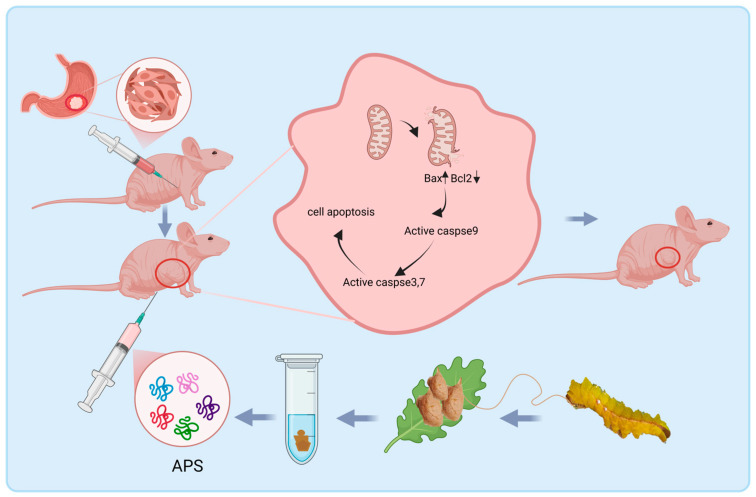
A schematic illustration of model regulation. APS reduces the mitochondrial membrane potential and activates the caspase-dependent mitochondrial apoptosis pathway, thus promoting apoptosis of GC cells.

In summary, we revealed that APS effectively suppressed GC growth and regulated the expression of Bcl2 and Bax to induce apoptosis, thus exhibiting anticancer activity (Figure 8). Moreover, it does not show discernible harm to gastric mucosal cells and mice, indicating that APS is a prospective curative agent for GC treatment, with low toxicity and few adverse side effects.

## 4. Materials and Methods

### 4.1. Sericin Extraction

Cocoons of *Bombyx mori* (871 strain), *Antherea pernyi* (Yuda1 strain), and *Bombyx mandarina* (AK18 strain) were obtained from the State Key Laboratory of Resource Insects, Henan Research Academy of Sericultural Science, and Shaanxi Key Laboratory of Sericulture, Ankang University, respectively. Sericin is easily soluble in water and alkaline solutions, whereas fibroin is insoluble in water and most organic solvents and requires special solvents such as LiBr, formic acid, HFIP (hexafluoroisopropanol), or a CaCl_2_/ethanol/water system for dissolution. Therefore, all the cocoon pieces were boiled in 1% sodium carbonate solution for 1.5 h and centrifuged at 10,000× *g* for 10 min, and then the supernatant was collected. The filtrate was dialyzed against MiliQ H_2_O with a molecular weight cutoff of 8 kDa (Solarbio, Beijing, China) for 72 h. The dialyzed protein solution was freeze-dried and stored at −80 °C. After sericin extraction, the remaining extract was washed with ddH_2_O and dried for weighing to evaluate the degumming rate. Typically, after degumming 50 g of cocoons using this method, the remaining material weighs approximately 47 g, resulting in a degumming rate of around 6%.

### 4.2. Ultraviolet Absorption Spectrum Detection

Equal amounts of three kinds of sericin were dissolved in the same volume of ddH_2_O, and after complete dissolution, the UV absorption spectra of the three kinds of sericin were detected by a spectrophotometer (SHIMADZU, Shanghai, China), and the ddH_2_O was adjusted to zero.

### 4.3. Cell Culture

Human GES-1, MKN45, and AGS cells were obtained from the American Type Culture Collection (ATCC, Manassas, VA, USA). These cells were cultured in RPMI-1640 medium (Gibco, New York, NY, USA) supplemented with 100 U/mL penicillin, 100 μg/mL streptomycin, and 10% fetal bovine serum (BIOAGRIO, Swampscott, MA, USA). MKN45/CDDP cells were obtained from Zhejiang Matson Cell Technology Co., Ltd. (Huzhou, China), and cultured in RPMI-1640 complete medium with 1 μM cisplatin (Sigma, St. Louis, MO, USA). The cell line was cultivated in an incubator (Thermo Fisher, Waltham, MA, USA) with 5% carbon dioxide (CO_2_) at 37 °C. The sericin protein is completely soluble in water and can directly dissolve in the medium during subsequent cell treatment.

### 4.4. CCK-8 Assay

The Cell Counting Kit-8 (CCK-8, MCE, Buford, GA, USA) was used to determine the viability of the cells after they were seeded into 96-well plates (Corning, New York, NY, USA) and grown at 37 °C with 5% CO_2_. The Multifunctional Enzyme Marker (BioTek, Winooski, VT, USA) was used to measure the absorbance value at 450 nm. All experiments had three replicates.

### 4.5. Apoptosis Detection

The Annexin V-FITC apoptosis detection kit (Beyotime, Shanghai, China) was used for flow cytometry analysis. The cells were centrifuged at 1000× *g* for 5 min, and washed with phosphate-buffered saline (PBS, Solarbio, Beijing, China). After that, the cells were stained with PI and Annexin V-FITC at room temperature for 20 min. Finally, a CytoFLEX flow cytometer (Beckman Coulter Biotech, Shanghai, China) was used to detect the cells. Positive FITC staining and negative PI staining showed early apoptotic cells; positivity for both stains indicated late apoptotic cells.

The TUNEL stain experiment was performed using the one-step TUNEL cell apoptosis detection kit (Beyotime, Shanghai, China). Cells were seeded in a 24-well plate (Corning, New York, NY, USA) with slips (Fisher Scientific, Waltham, MA, USA). For the analysis, the cell culture medium was removed, and the cells were fixed with 4% paraformaldehyde (Solarbio, Beijing, China) for 30 min using 0.1% Triton X-100 permeabilization for 10 min. After that, the cells were washed with PBS, and 50 μL of the TUNEL test solution was added. The cells were incubated at 37 °C for 60 min without light and then washed with PBS. Finally, the cells were imaged using a laser scanning confocal microscope (Olympus, Center Valley, PA, USA).

The caspase enzymatic activity was tested using the Caspase Glo^®^ 3/7 Assay System and the Caspase Glo^®^ 9 Assay System (Promega, Beijing, China). A volume of 50 μL of digested cell suspension was added to a 96-well enzyme plate (Corning, New York, NY, USA), and 50 μL of Caspase 3/7 or Caspase 9 assay reagent was added. Then, the cells were incubated at 37 °C for 1 h. The luminescence value was measured using the multifunctional enzyme marker (BioTek, Winooski, VT, USA).

### 4.6. Plate Colony Formation Assay

Cells were cultured in plates for 10 days. After that, the medium was removed, cells were washed with PBS 2 times, and then 4% paraformaldehyde was used for immobilization for 15 min. Finally, cells were stained with 0.1% crystal violet solution for 10 min and then washed. Cell colony formation was analyzed using the ImageJ 1.53k software.

### 4.7. Transwell Migration Assay

For the Transwell migration experiment, 5 × 10^4^ cells were seeded in the upper chamber (Corning, New York, NY, USA) with serum-free medium, 600 μL of medium containing 20% fetal bovine serum was added to the lower chamber; cells were cultivated for 48 h at 37 °C in a cell incubator. After discarding the medium, a cotton swab was used to clean the cells in the upper chamber. Then, the cells were fixed with 4% paraformaldehyde for 30 min, after which they were stained with a 0.1% crystal violet solution for 10 min, and washed with PBS. Finally, the chambers were imaged using an electron microscope (Olympus, Center Valley, PA, USA). The results were analyzed using the ImageJ software.

### 4.8. Mitochondrial Membrane Potential Detection

The mitochondrial membrane potential assay kit with JC-1 (Beyotime, Shanghai, China) was used in accordance with the manufacturer’s instructions. The steps were as follows: the culture medium was removed from the cells and discarded, and JC-1 dye working fluid was added; the cells were incubated for 20 min at 37 °C. After that, the cells were washed with JC-1 buffer 3 times. The mitochondrial membrane potential was measured using a laser scanning confocal microscope or flow cytometer.

### 4.9. Toxicological Testing

A toxicological assay was performed on 6-week-old C57BL/6 female mice weighing 16–18 g. The mice were obtained from ENSIWEIER Biotechnology Co., Ltd. (Shanghai, China). For 14 days, the mice were given PBS or 5000 mg/kg or 10,000 mg/kg sericin intragastrically. Body weight fluctuations were recorded over the course of treatment. The Southwest University Animal Care Committee approved the experimental design of this study.

### 4.10. Tumor Xenograft Assay

A tumor xenografts assay was performed on 4-week-old BALB/c nude female mice (Charles River Laboratory Animal Technology Co., Ltd., Beijing, China). The mice were fed in rooms free of pathogens. MKN45 cells (1 × 10^6^) were implanted subcutaneously into their underarm. The sericin group mice received an intraperitoneal injection of sericin (500 mg/kg) for 14 days, and the control group was injected with ddH_2_O. The tumor volume was measured using the following equation: volume = (length × width^2^)/2. At the end of the experiment, tumors were harvested from the mice and weighed. The Southwest University Animal Care Committee approved the experimental design of this study.

### 4.11. Hematoxylin and Eosin (H&E) Staining Assay

The pathological structures of the mouse lung, spleen, kidney, heart, liver, and stomach were assessed using an H&E staining kit (Lelai, Shanghai, China). Samples of the tissues were fixed in 4% paraformaldehyde, dried, embedded, and cut into sections. After being deparaffinized and rehydrated, the sections were stained for 24 h at 4 °C using H&E staining buffer. Lastly, the sections were observed under an electron microscope.

### 4.12. Quantitative Real-Time (qRT)-PCR

The Total RNA Kit (Omega Bio-Tek, Norcross, GA, USA) was used to extract the total RNA from cells. Then, RNA was reversed-transcribed into cDNA using the Prime Script™ RT Reagent Kit (TaKaRa, Beijing, China). qRT-PCR was carried out with Hieff^®^ qPCR SYBR Green Master Mix (Yeasen, Shanghai, China). The parameters for the reaction were 95 °C for 30 s, 40 cycles of 95 °C for 5 s, and 60 °C for 30 s, with three repetitions for each sample. The internal reference gene was *β*-actin. The primers are shown in Table S1.

### 4.13. Western Blot Analysis

The cells treated with APS (4 mg/mL) for 48 h. Then, the Western lysis buffer (Beyotime, Shanghai, China) was added to the cells. After 2 h, the cells were centrifuged at 13,000× *g* for 10 min, and the protein supernatant was collected. Proteins were mixed with 5 × SDS loading buffer (Beyotime, Shanghai, China) and boiled for 10 min. After SDS-PAGE gel electrophoresis, the denatured protein was transferred to a polyvinylidene fluoride (PVDF) membrane (Millipore, Burlington, MA, USA), which was then blocked with 5% skim milk powder, incubated with the Bcl2, Bax, or Tubulin antibody (Proteintech, Wuhan, China) for 2 h, and then washed with TBST 6 times. Then, the membrane was incubated with the secondary antibody (Beyotime, Shanghai, China) for 1 h. Finally, the Western blot results were analyzed with the ECL Western blotting Detection System (Bio-Rad, Hercules, CA, USA).

### 4.14. Statistical Analysis

GraphPad Prism 6 (GraphPad, La Jolla, CA, USA) was used for statistical analysis. The significance of the variations between treatment groups was assessed using the *t*-test. *p* < 0.05 indicated significant differences, and *p* < 0.01 indicated extremely significant differences. The mean ± SD of a minimum of three separate biological replicates is used to show the data.

## Data Availability

Data are contained within the article and Supplementary Materials.

## Supplementary Materials

The supporting information can be downloaded at https://www.mdpi.com/article/10.3390/ijms26051890/s1.

## References

[1] Bray F., Laversanne M., Sung H., Ferlay J., Siegel R.L., Soerjomataram I., Jemal A. (2024). Global cancer statistics 2022: GLOBOCAN estimates of incidence and mortality worldwide for 36 cancers in 185 countries. CA Cancer J. Clin..

[2] Xia C., Dong X., Li H., Cao M., Sun D., He S., Yang F., Yan X., Zhang S., Li N. (2022). Cancer statistics in China and United States, 2022: Profiles, trends, and determinants. Chin. Med. J..

[3] Luo G., Zhang Y., Guo P., Wang L., Huang Y., Li K. (2017). Global patterns and trends in stomach cancer incidence: Age, period and birth cohort analysis. Int. J. Cancer.

[4] Bhandare M.S., Gundavda K.K., Yelamanchi R., Chopde A., Batra S., Kolhe M., Ramaswamy A., Ostwal V., Deodhar K., Chaudhari V. (2024). Impact of pCR after neoadjuvant chemotherapy and radical D2 dissection in locally advanced gastric cancers: Analysis of 1001 cases. Eur. J. Surg. Oncol. J. Eur. Soc. Surg. Oncol. Br. Assoc. Surg. Oncol..

[5] Manzoor M., Singh J., Gani A. (2022). Exploration of bioactive peptides from various origin as promising nutraceutical treasures: In vitro, in silico and in vivo studies. Food Chem..

[6] Mansourian M., Badiee A., Jalali S.A., Shariat S., Yazdani M., Amin M., Jaafari M.R. (2014). Effective induction of anti-tumor immunity using p5 HER-2/neu derived peptide encapsulated in fusogenic DOTAP cationic liposomes co-administrated with CpG-ODN. Immunol. Lett..

[7] Russo L.C., Araujo C.B., Iwai L.K., Ferro E.S., Forti F.L. (2017). A Cyclin D2-derived peptide acts on specific cell cycle phases by activating ERK1/2 to cause the death of breast cancer cells. J. Proteom..

[8] Adams J.M. (2012). Therapeutic potential of a peptide targeting BCL-2 cell guardians in cancer. J. Clin. Investig..

[9] Masuzawa T., Fujiwara Y., Okada K., Nakamura A., Takiguchi S., Nakajima K., Miyata H., Yamasaki M., Kurokawa Y., Osawa R. (2012). Phase I/II study of S-1 plus cisplatin combined with peptide vaccines for human vascular endothelial growth factor receptor 1 and 2 in patients with advanced gastric cancer. Int. J. Oncol..

[10] Fu X.Y., Yin H., Chen X.T., Yao J.F., Ma Y.N., Song M., Xu H., Yu Q.Y., Du S.S., Qi Y.K. (2024). Three Rounds of Stability-Guided Optimization and Systematical Evaluation of Oncolytic Peptide LTX-315. J. Med. Chem..

[11] Yin H., Chen X.T., Chi Q.N., Ma Y.N., Fu X.Y., Du S.S., Qi Y.K., Wang K.W. (2023). The hybrid oncolytic peptide NTP-385 potently inhibits adherent cancer cells by targeting the nucleus. Acta Pharmacol. Sin..

[12] Feng J., Xiao T., Lu S.S., Hung X.P., Yi H., He Q.Y., Huang W., Tang Y.Y., Xiao Z.Q. (2020). ANXA1-derived peptides suppress gastric and colon cancer cell growth by targeting EphA2 degradation. Int. J. Oncol..

[13] Zhou D., Liu W., Liang S., Sun B., Liu A., Cui Z., Han X., Yuan L. (2018). Apoptin-derived peptide reverses cisplatin resistance in gastric cancer through the PI3K-AKT signaling pathway. Cancer Med..

[14] Hong S.M., Choi S.C., Park H.M., Seok Y.S. (2019). Preparation and characterization of sericin powder extracted with deep sea water. 3 Biotech..

[15] Lamboni L., Gauthier M., Yang G., Wang Q. (2015). Silk sericin: A versatile material for tissue engineering and drug delivery. Biotechnol. Adv..

[16] Kumar J.P., Mandal B.B. (2017). Antioxidant potential of mulberry and non-mulberry silk sericin and its implications in biomedicine. Free Radic. Biol. Med..

[17] Joyjamras K., Chaotham C., Chanvorachote P. (2022). Response surface optimization of enzymatic hydrolysis and ROS scavenging activity of silk sericin hydrolysates. Pharm. Biol..

[18] Cao T.T., Zhang Y.Q. (2016). Processing and characterization of silk sericin from Bombyx mori and its application in biomaterials and biomedicines. Mater. Sci. Eng. C Mater. Biol. Appl..

[19] Aramwit P., Damrongsakkul S., Kanokpanont S., Srichana T. (2010). Properties and antityrosinase activity of sericin from various extraction methods. Biotechnol. Appl. Biochem..

[20] Khosropanah M.H., Vaghasloo M.A., Shakibaei M., Mueller A.L., Kajbafzadeh A.M., Amani L., Haririan I., Azimzadeh A., Hassannejad Z., Zolbin M.M. (2022). Biomedical applications of silkworm (Bombyx Mori) proteins in regenerative medicine (a narrative review). J. Tissue Eng. Regen. Med..

[21] Kunz R.I., Brancalhão R.M., Ribeiro L.F., Natali M.R. (2016). Silkworm Sericin: Properties and Biomedical Applications. BioMed Res. Int..

[22] Chen Z., He Y., Song C., Dong Z., Su Z., Xue J. (2012). Sericin can reduce hippocampal neuronal apoptosis by activating the Akt signal transduction pathway in a rat model of diabetes mellitus. Neural Regen. Res..

[23] Kumar J.P., Mandal B.B. (2019). Silk sericin induced pro-oxidative stress leads to apoptosis in human cancer cells. Food Chem. Toxicol..

[24] Niu L., Yang S., Zhao X., Liu X., Si L., Wei M., Liu L., Cheng L., Qiao Y., Chen Z. (2021). Sericin inhibits MDA-MB-468 cell proliferation via the PI3K/Akt pathway in triple-negative breast cancer. Mol. Med. Rep..

[25] Kaewkorn W., Limpeanchob N., Tiyaboonchai W., Pongcharoen S., Sutheerawattananonda M. (2012). Effects of silk sericin on the proliferation and apoptosis of colon cancer cells. Biol. Res..

[26] Zhaorigetu S., Yanaka N., Sasaki M., Watanabe H., Kato N. (2003). Silk protein, sericin, suppresses DMBA-TPA-induced mouse skin tumorigenesis by reducing oxidative stress, inflammatory responses and endogenous tumor promoter TNF-alpha. Oncol. Rep..

[27] Ji L., Liu Z., Zhou B., Cai Y., An F., Wang L., Lv Z., Xia M., Yang J., Yuan J. (2020). Community-Based Pilot Study of a Screening Program for Gastric Cancer in a Chinese Population. Cancer Prev. Res..

[28] Kumar Dan A., Aamna B., De S., Pereira-Silva M., Sahu R., Cláudia Paiva-Santos A., Parida S. (2022). Sericin nanoparticles: Future nanocarrier for target-specific delivery of chemotherapeutic drugs. J. Mol. Liq..

[29] Aramwit P., Kanokpanont S., De-Eknamkul W., Srichana T. (2009). Monitoring of inflammatory mediators induced by silk sericin. J. Biosci. Bioeng..

[30] Kato N., Sato S., Yamanaka A., Yamada H., Fuwa N., Nomura M. (1998). Silk protein, sericin, inhibits lipid peroxidation and tyrosinase activity. Biosci. Biotechnol. Biochem..

[31] Aramwit P., Napavichayanum S., Pienpinijtham P., Rasmi Y., Bang N. (2020). Antibiofilm activity and cytotoxicity of silk sericin against Streptococcus mutans bacteria in biofilm: An in vitro study. J. Wound Care.

[32] Zhou B., Wang H. (2020). Structure and Functions of Cocoons Constructed by Eri Silkworm. Polymers.

[33] Aramwit P., Sangcakul A. (2007). The effects of sericin cream on wound healing in rats. Biosci. Biotechnol. Biochem..

[34] Elahi M., Ali S., Tahir H.M., Mushtaq R., Bhatti M.F. (2020). Sericin and fibroin nanoparticles—Natural product for cancer therapy: A comprehensive review. Int. J. Polym. Mater. Polym. Biomater..

[35] Ma Y., Zhou L., Yang C., Wang L., Yi S., Tong X., Xiao B., Chen J. (2021). Comparison of Sericins from Different Sources as Natural Therapeutics against Ulcerative Colitis. ACS Biomater. Sci. Eng..

[36] Silva S.S., Kundu B., Lu S., Reis R.L., Kundu S.C. (2019). Chinese Oak Tasar Silkworm Antheraea pernyi Silk Proteins: Current Strategies and Future Perspectives for Biomedical Applications. Macromol. Biosci..

[37] Chen R., Zhu C., Hu M., Zhou L., Yang H., Zheng H., Zhou Y., Hu Z., Peng Z., Wang B. (2019). Comparative analysis of proteins from Bombyx mori and Antheraea pernyi cocoons for the purpose of silk identification. J. Proteom..

[38] Dai Z.J., Sun W., Zhang Z. (2019). Comparative analysis of iTRAQ-based proteomes for cocoons between the domestic silkworm (*Bombyx mori*) and wild silkworm (*Bombyx mandarina*). J. Proteom..

[39] Elmore S. (2007). Apoptosis: A review of programmed cell death. Toxicol. Pathol..

[40] Zou Y., Li Q., Jiang L., Guo C., Li Y., Yu Y., Li Y., Duan J., Sun Z. (2016). DNA Hypermethylation of CREB3L1 and Bcl-2 Associated with the Mitochondrial-Mediated Apoptosis via PI3K/Akt Pathway in Human BEAS-2B Cells Exposure to Silica Nanoparticles. PLoS ONE.

[41] Smyth E.C., Nilsson M., Grabsch H.I., van Grieken N.C., Lordick F. (2020). Gastric cancer. Lancet.

[42] Bubley G.J., Xu J., Kupiec N., Sanders D., Foss F., O’Brien M., Emi Y., Teicher B.A., Patierno S.R. (1996). Effect of DNA conformation on cisplatin adduct formation. Biochem. Pharmacol..

[43] Siddik Z.H. (2003). Cisplatin: Mode of cytotoxic action and molecular basis of resistance. Oncogene.

[44] Marullo R., Werner E., Degtyareva N., Moore B., Altavilla G., Ramalingam S.S., Doetsch P.W. (2013). Cisplatin induces a mitochondrial-ROS response that contributes to cytotoxicity depending on mitochondrial redox status and bioenergetic functions. PLoS ONE.

[45] Sorenson C.M., Eastman A. (1988). Mechanism of cis-diamminedichloroplatinum(II)-induced cytotoxicity: Role of G2 arrest and DNA double-strand breaks. Cancer Res..

[46] Oliveira C.A., Mercês É.A.B., Portela F.S., Malheiro L.F.L., Silva H.B.L., De Benedictis L.M., De Benedictis J.M., Silva C., Santos A.C.L., Rosa D.P. (2024). An integrated view of cisplatin-induced nephrotoxicity, hepatotoxicity, and cardiotoxicity: Characteristics, common molecular mechanisms, and current clinical management. Clin. Exp. Nephrol..

[47] Giovanni M., Tay C.Y., Setyawati M.I., Xie J., Ong C.N., Fan R., Yue J., Zhang L., Leong D.T. (2015). Toxicity profiling of water contextual zinc oxide, silver, and titanium dioxide nanoparticles in human oral and gastrointestinal cell systems. Environ. Toxicol..

[48] Chlapanidas T., Faragò S., Lucconi G., Perteghella S., Galuzzi M., Mantelli M., Avanzini M.A., Tosca M.C., Marazzi M., Vigo D. (2013). Sericins exhibit ROS-scavenging, anti-tyrosinase, anti-elastase, and in vitro immunomodulatory activities. Int. J. Biol. Macromol..

[49] Kundu S.C., Dash B.C., Dash R., Kaplan D.L. (2008). Natural protective glue protein, sericin bioengineered by silkworms: Potential for biomedical and biotechnological applications. Prog. Polym. Sci..

[50] Sasaki M., Yamada H., Kato N. (2000). Consumption of silk protein, sericin elevates intestinal absorption of zinc, iron, magnesium and calcium in rats. Nutr. Res..

