# Supplementary material for: The Natural Antherea pernyi Sericin Protein Suppresses Gastric Cancer Formation by Inhibiting Cell Proliferation and Inducing Cell Apoptosis
Source: Int J Mol Sci. 2025 Feb 22;26(5):1890. doi: 10.3390/ijms26051890 (PMC11900216; doi:10.3390/ijms26051890)
Supplement: Supplementary file 1 [file ijms-26-01890-s001.zip › ijms-3451995-supplementary.pdf]

Supplementary Materials:

Directory of graph

Serial number	Name
Figure 1	The effects of sericin from different sources on the apoptosis, proliferation, and oxidation of cells
Figure 2	Safety evaluation of APS.
Figure 3	The effects of APS on GC cells proliferation, clonogenicity, and migration.
Figure 4	The effects of APS on GC cell apoptosis.
Figure 5	Antitumorigenic effects of APS on the GC.
Figure 6	The effects of APS on apoptosis in MKN45/CDDP cells.
Figure 7	The effects of APS with cisplatin on proliferation and apoptosis in GC cells.
Figure 8	A schematic illustration of model regulation.
Figure S1	The peroxidation model of GES1 cells was established.
Figure S2	Safely evaluation of APS
Figure S3	APS had no effect on GES1 cells.
Figure S4	APS suppresses tumorigenesis of GC cells.
Figure S5	APS has no synergistic effect with cisplatin.

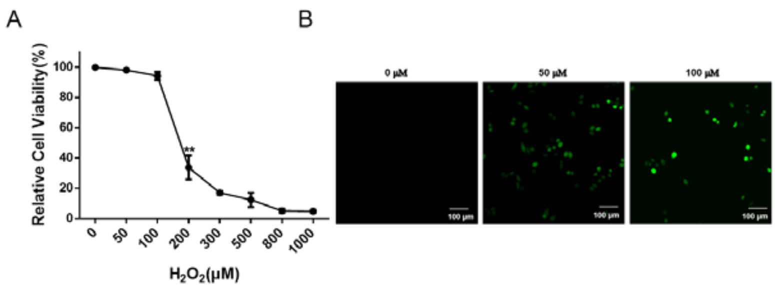

Figure S1. The peroxidation model of GES1 cells was established. (A) The cell viability of GSE1 cells treated with various concentrations of H_2O_2 (50 μM, 100μM, 200 μM, 300 μM, 500 μM, 800 μM and 1000 μM) for 12 h via CCK-8 assay. **, *p*<0.01. (B) The ROS levels of GES1 cells were measured by DCFH-DA after H_2O_2 treated (0, 50 and 100 μM, respectively) for 12 h. Scale bar = 100 μm.

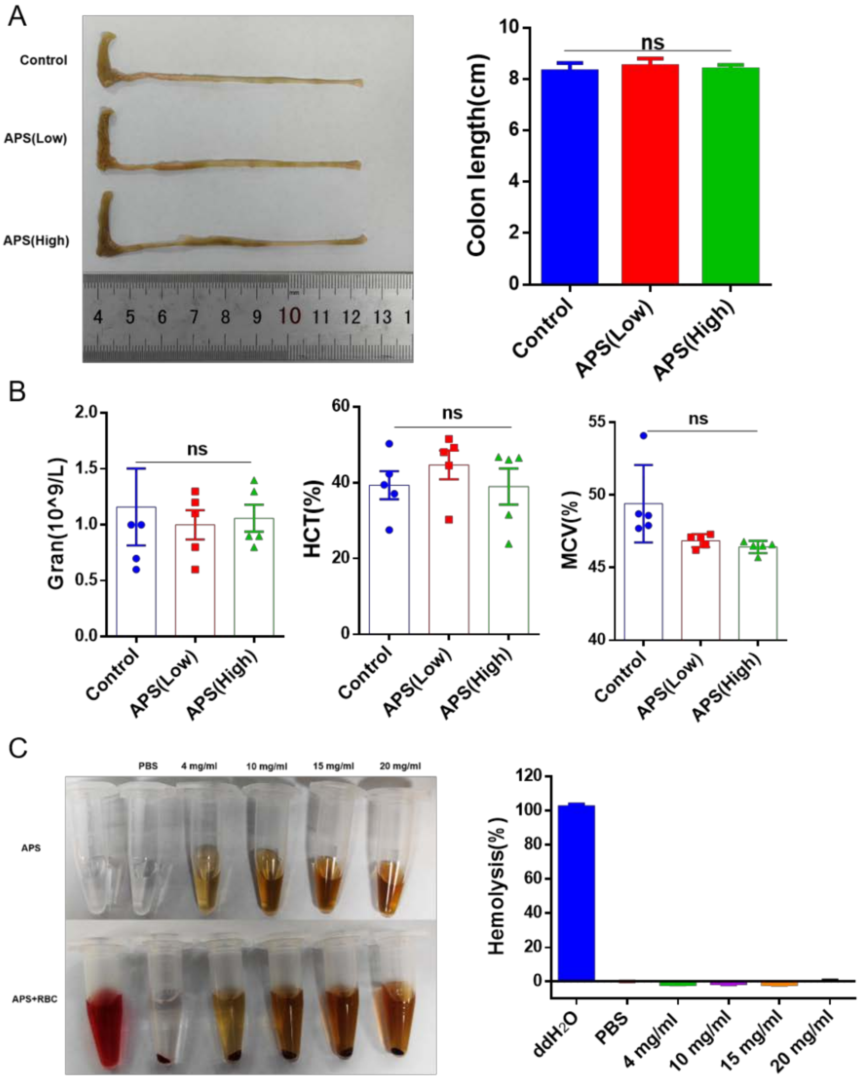

Figure S2. Safely evaluation of APS. (A) The colonic length of in mice. (B) The examination of routine blood parameters, including monocyte (Gran), hematocrit (HCT), mean erythrocyte volume (MCV). (C) Effects of APS with different concentration on hemolysis, the negative control is PBS, positive control is ddH_2O. ns, *p*>0.05.

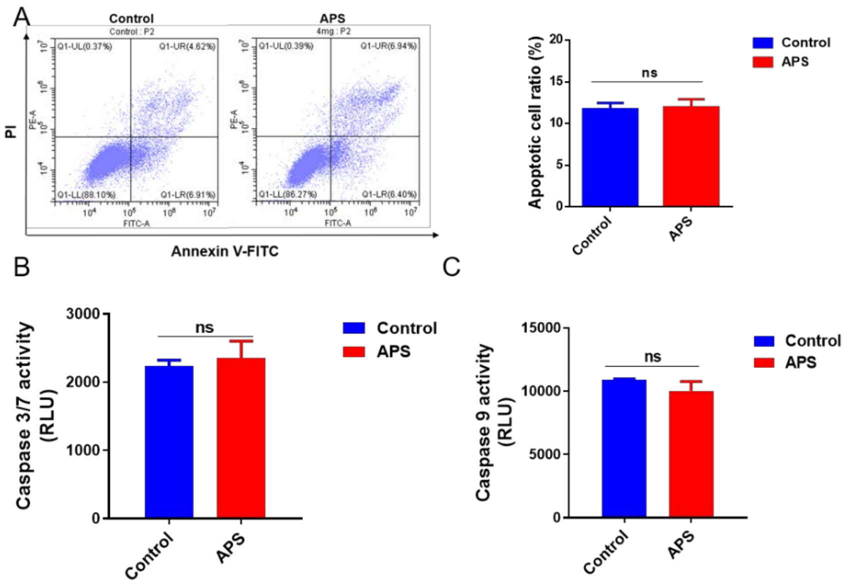

Figure S3. APS had no effect on GES1 cells. (A) The level of apoptosis was determined via flow cytometry after GES1 cells were treated for 24 h with APS (4 mg/mL). (B, C) Caspase 3/7 and caspase 9 enzyme activities were measured to evaluate the impact of APS on apoptosis. ns, *p*>0.05.

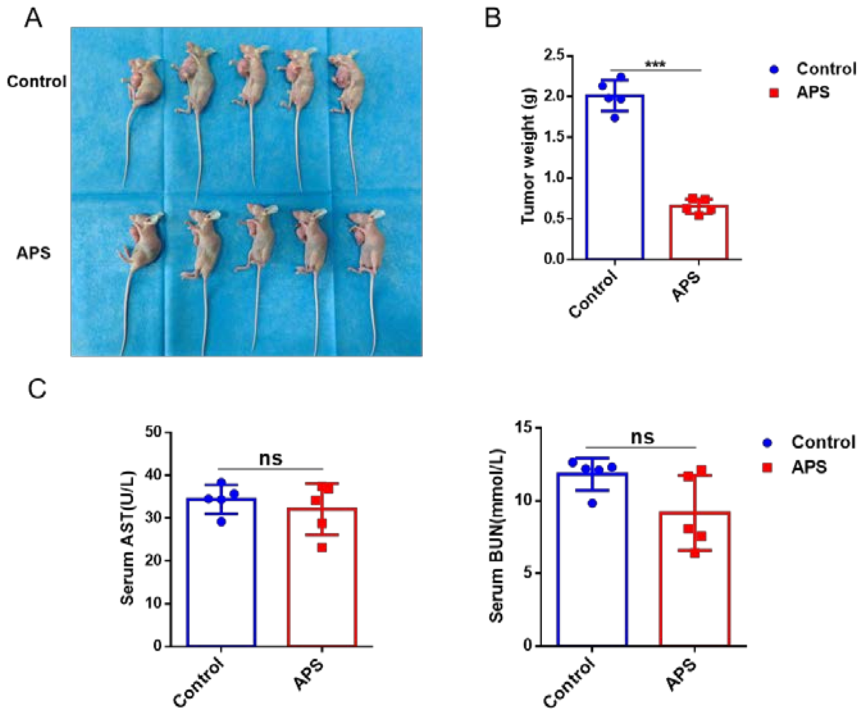

Figure S4. APS suppresses tumorigenesis of GC cells. (A) Tumor xenografts were tested in MKN45 cells after treating with APS (500 mg/kg) or ddH_2O (Control), (n = 5). (B) The tumor weight of mice. (C) Serum markers of liver and kidney function: AST and BUN. ***, *p*<0.001; and ns, *p*>0.05.

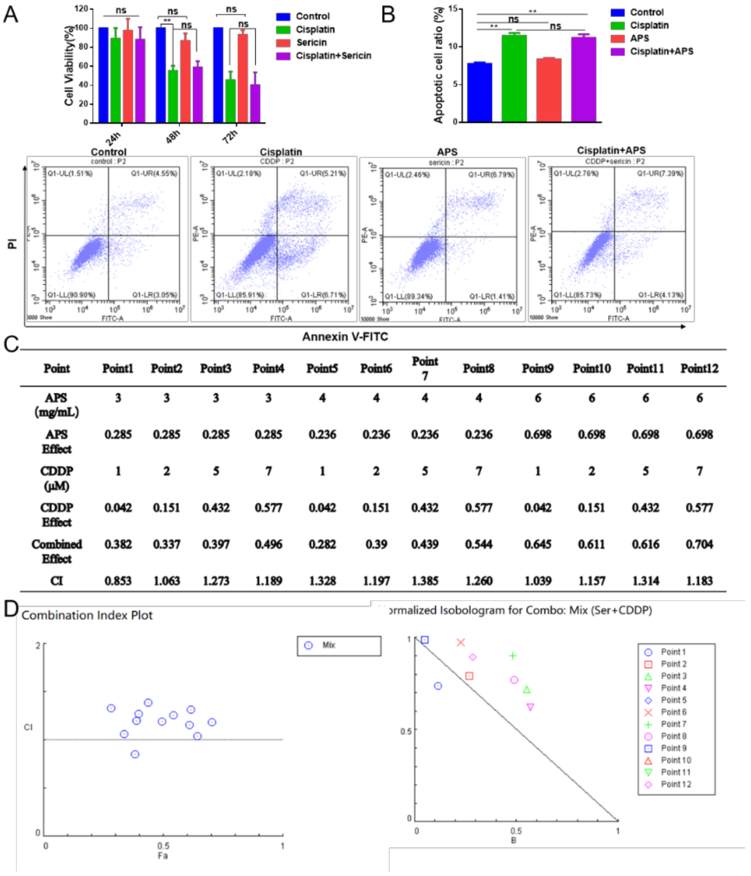

Point	Point1	Point2	Point3	Point4	Point5	Point6	Point 7	Point8	Point9	Point10	Point11	Point12
APS (mg/mL)	3	3	3	3	4	4	4	4	6	6	6	6
APS Effect	0.285	0.285	0.285	0.285	0.236	0.236	0.236	0.236	0.698	0.698	0.698	0.698
CDDP (μM)	1	2	5	7	1	2	5	7	1	2	5	7
CDDP Effect	0.042	0.151	0.432	0.577	0.042	0.151	0.432	0.577	0.042	0.151	0.432	0.577
Combined Effect	0.382	0.337	0.397	0.496	0.282	0.39	0.439	0.544	0.645	0.611	0.616	0.704
CI	0.853	1.063	1.273	1.189	1.328	1.197	1.385	1.260	1.039	1.157	1.314	1.183

Figure S5. APS has no synergistic effect with cisplatin. (A) The cell viability of GES1cells after treating with APS (4 mg/ml) and different concentrations of cisplatin for 24 h was tested by CCK-8. (B) The GES1 cells apoptosis was evaluated by flow cytometry analysis after APS (4 mg/mL) or/and cisplatin (5 μM) treated for 24 h. (C) APS and cisplatin concentration Settings, combined effects and CI values by CompuSyn software; (D) CI value and the combined effect scatter plot, dose normalized isoeffect plots of the two drugs, with cisplatin as the ordinate and APS as the abscissa. **, *p*<0.01; and ns, *p*>0.05.

Tabel S1 Sequences and names of primers

Tabel S1 Sequences and names of primers

Primer	Sequence
Bax-F	CCTGTGCACCAAGGTGCCGGAACT
Bax-R	CCACCCTGGTCTTGGATCCAGCCC
Bcl-2-F	GTGGATGACTGAGTACCTGAAC
Bcl-2-R	GCCAGGAGAAATCAAACAGAGG

Caspase3-F	ACATGGCGTGTCATAAAATACC
Caspase3-R	CACAAAGCGACTGGATGAAC
Caspase9-F	AGTTCCCGGGTGCTGTCTAT
Caspase9-R	GCCATGGTCTTTCTGCTCAC
β-actin-F	CCAACCGCGAGAAGATGA
β-actin-R	CCAGAGGCGTACAGGGATAG
